# Complexity of Medicine Regimens and Patient Perception of Medicine Burden

**DOI:** 10.3390/pharmacy7010018

**Published:** 2019-02-02

**Authors:** Janet Krska, Sarah A. Corlett, Barbra Katusiime

**Affiliations:** Medway School of Pharmacy, Universities of Greenwich and Kent, Chatham Maritime ME4 4TB, Kent, UK; s.a.corlett@kent.ac.uk (S.A.C.); b.katusiime@kent.ac.uk (B.K.)

**Keywords:** medicine burden, regimen complexity, formulation, patient perception

## Abstract

From the patient perspective, medicine burden is more than the number of medicines, or the complexity of medicine regimens they need to manage. Relationships between the number of medicines, regimen complexity and patient perception of medicine burden are under-researched. This cross-sectional study measured regimen complexity and determined how this and patient perceived burden are affected by the therapeutic group. Regimen complexity was measured in patients presenting prescriptions to six community pharmacies in South-East England. A sub-sample (166) also completed the Living with Medicines Questionnaire which measures patient perceived burden. The 492 patients were prescribed 2700 medicines (range 1 to 23). Almost half used at least one non-oral formulation. Complexity was correlated strongly with the number of medicines (r = 0.94), number of therapeutic groups (r = 0.84) and number of formulations (r = 0.73). Patients using medicines for skin, eye and respiratory conditions had the highest complexity scores. Increasing the number of medicines, frequency of dosing, number of non-oral formulations and number of different therapeutic groups all increased medicine burden. Although cardiovascular medicines were the most common medicines used by the majority of patients (60%), those for neurological, psychiatric and gastro-intestinal conditions were most strongly associated with high burden. Studies are required to determine medicine burden in different conditions, especially neurological conditions, including chronic pain.

## 1. Introduction

While the increasing number of prescription medicines required to manage chronic conditions is acknowledged, the term medicine burden is used differently by different researchers. The biomedical perspective of medicine burden is often interpreted simply as the number of medicines or daily “pills” individual patients need to take to manage their conditions. Many studies have used the term medicine burden to describe the number of medicines used by specific cohorts within populations [1,2,3,4]. Such studies suggest that a higher medicine burden may exist in certain medical conditions, for example a study in Sweden suggests that patients with type II diabetes use more medicines than those without [1].

However, medicines are more complex than this, involving a wide range of formulations with varying instructions, requiring different degrees of effort on the part of the patient to use correctly. The difficulties patients experience in using medicines may vary with both the formulation and the frequency and timing of dosing. A method of measuring the complexity of medicine regimens is available, the Medication Regimen Complexity Index (MRCI) [5]. This instrument can be applied to prescription data and could provide a more detailed measure of burden, than simply the number of medicines, although MRCI score correlates highly with the number of medicines. The MRCI gives higher weightings to formulations with greater difficulty in administration and to more frequent administration. Therefore, applying the MRCI requires full information about all the medicines prescribed for an individual patient, including total number, dosage forms, dosing frequency and additional directions. Although data are widely available on overall medicines prescribed in England [6], relatively little data are available on the number of medicines used by individuals and even less is known about the complexity of individual regimens. Data from Scotland published in 2014 found that 16.9% of adults were receiving four to nine medicines with 4.6% having ten or more [7]. A more recent English study involving 5213 patients over 60 years of age reported that almost a third were using five or more medicines regularly, and only a fifth used none [8]. We found no studies which measured regimen complexity in an English population.

The term burden has also been widely used in connection with medicines with anticholinergic effects. A number of anticholinergic burden measures have been developed and used particularly to assess cognitive function [9]. Most show that higher anticholinergic burden is associated with poorer cognitive and functional outcomes. 

However, all these measures of medicine burden are obtained from prescription details and do not consider the impact of medicine use on the patient, from their perspective. More recently, an increasing number of studies are exploring medicine burden from the patient perspective [10]. Medicine burden is a large part of overall treatment burden, and given that medicines are the main method of treatment for most medical conditions, they are the component of treatment burden most frequently identified in qualitative studies [11]. These authors argue that treatment burden is associated with demands placed upon patients, which is influenced by the number of medical conditions and the number of medicines. They found that patients routinize and integrate their treatments into their daily lives, perhaps prioritizing some over others, as well as using resources, such as social support, to help manage their treatment burden [11]. Given the magnitude of medicines as treatments, the patient perspective of medicine burden merits separate investigation from treatment burden. Medicine burden from the patient perspective is not simply the number of medicines, or the complexity of medicine regimens, but encompasses a range of domains. 

We have developed and validated an instrument for measuring medicine burden: The Living with Medicines Questionnaire (LMQ) [12,13]. The LMQ version 3 (LMQ-3) covers eight domains which could be burdensome to individuals: relationships with health professionals, practicalities, interferences, effectiveness, side effects, concerns, cost and autonomy. Our data suggest that patients prescribed a higher number of medicines, using non-oral and multiple formulations and with multiple daily dosage regimens perceive higher medicine burden [13]. However, medicine complexity, as measured by the MRCI, is only weakly correlated with medicine burden [14,15]. Therefore, we set out to explore this association in greater detail. Our objectives were to measure the complexity of prescribed medicine regimens in a sample of English patients and assess whether the therapeutic group affects regimen complexity; to measure the self-reported burden of using long-term medicines in this population and any associations with the medicine formulation, dosing frequency and therapeutic group.

## 2. Materials and Methods 

This cross-sectional survey involved patients presenting prescriptions to one of six community pharmacies in the county of Kent, England, during October/November 2016. NHS Ethics approval was obtained (Ref number: 16/YH/0413).

### 2.1. Inclusion Criteria

- Adults aged 18 years old or over
- Resident in England
- Using at least one long-term prescription medicine for at least 6 months
- Able to understand and read English

### 2.2. Exclusion Criteria

- Under 18 years of age
- Not resident in England
- Presenting a prescription for another person
- Use of regular prescription medicine for less than 6 months
- Unable to understand English sufficiently to give informed consent or complete the questionnaire

A small multiple pharmacy company owning over 100 pharmacies in southeast England supported the study. Six community pharmacies were selected to ensure a mix of deprivation and the responsible pharmacist in each agreed to their pharmacy being included. In each pharmacy, patients who had presented a prescription for dispensing were approached to take part by one of six student researchers trained to ensure uniformity of approach. The study was explained verbally and, if interested, screening questions were asked to ensure eligibility. Those who agreed were provided with the LMQ-3 plus an envelope for its return and asked to complete a consent form, giving permission for an anonymized list of their current prescription medicines to be made available from the pharmacist’s electronic record. An information sheet was also provided, giving contact details and offering the option to withdraw their consent at a later date.

The consent forms and questionnaires were coded with patient ID numbers to facilitate the subsequent matching of data. On return of each completed consent form to the community pharmacist, the pharmacist retrieved a list of current medicines, anonymized the list and added the ID number. 

### 2.3. Instruments

The MRCI was applied to all individual retrieved prescription records, following the published instructions [5]. In addition to the number of medicines, this allows the calculation of the overall complexity score. Each item from each prescription was also categorized by the therapeutic class of the medicine and the formulation type.

The LMQ-3 contains 41 statements covering eight domains related to medicine burden, using a Likert-type scale with responses ranging from strongly agree to strongly disagree. The total LMQ-3 scores were calculated from the responses to these statements, using reverse scoring as required. The scores were then categorized into low, moderate and high burden [13].

### 2.4. Data Analysis

The completed LMQ responses were matched to the prescription medicines lists. Associations between variables were measured using Pearson’s or Spearman’s correlation coefficients, depending on the type of data. Analysis of variance was used to determine differences in MRCI score across differing levels of burden (low/moderate/high). Differences in LMQ scores in the presence or absence of major therapeutic groups were assessed using t-tests. Linear regression analysis was performed using regimen complexity (MRCI score) or perceived burden (LMQ score) as dependent variables, including the presence of a medicine from each of the eight major therapeutic groups as the independent variables. A significance level of *p* < 0.05 was accepted.

## 3. Results

### 3.1. Response Rates

In total, 776 patients were invited to participate and 582 (75.0%) agreed. Of the 582 participants, 521 (89.5%) consented to the pharmacist providing an anonymized copy of their prescription medication record (PMR). Of the 521 PMRs analysed, 29 were subsequently excluded as the record was incomplete or the patient was not using a long-term medicine. There were 268 (46.0%) patients who returned the LMQ, although only 166 had both completed all 41 statements and also given permission for the PMR to be provided. 

### 3.2. Medicines Prescribed

A total of 2700 medicines were prescribed for the 492 patients. The mean number of medicines per patient was 5.49 (standard deviation 4.12), with a range of 1 to 23. Cardiovascular medicines were the most frequently prescribed, followed by those acting on the central nervous system, gastro-intestinal and respiratory systems, with these four major groups together constituting over 70% of all prescribed medicines (Table 1). 

Over 60% of the 492 patients whose prescription records were reviewed were using at least one cardiovascular medicine and over half were using a central nervous system (CNS) medicine. The number of prescribed medicines per patient was the highest for cardiovascular, CNS and respiratory drugs, and these patients were prescribed a median of two medicines from these groups. Almost a third of patients (158; 32.1%) were prescribed medicines from four or more different major therapeutic areas. Eighty-three patients (16.9%) were prescribed medicines from three major therapeutic areas, 129 (24.8%) from two, and 121 patients (24.6%) used medicines from only one therapeutic area. 

Almost all patients used oral solid dose formulations (477; 97.0%), but half (245; 49.8%) used other formulations: a fifth used inhalers and more than a fifth used topical preparations (Table 2). While over half (52.8%) used only one formulation, mostly oral, 153 (31.1%) used two formulations and 79 (16.1%) used three or more different formulations, adding to the complexity of managing their regimens.

### 3.3. Overall Medicine Complexity

The mean MRCI score was 14.1, with a range of 2–74. There was a very strong positive correlation between the number of medicines and the MRCI score (Pearson’s r = 0.94), with higher MRCI scores indicating greater regimen complexity. Complexity also correlated strongly with the number of therapeutic groups of medicines patients used (Spearman’s r = 0.84; *p* < 0.001) and the number of formulations used (r = 0.73). The mean MRCI scores were the highest in patients taking drugs for eye (27.0; n = 33), skin (23.8; n = 78) and respiratory conditions (23.7; n = 107). Patients taking drugs for cardiovascular conditions had the lowest mean MRCI scores (16.0; n = 298).

Linear regression analysis suggested that all major therapeutic groups contributed significantly to the MRCI score (Table 3). The number of medicines prescribed within the therapeutic groups all showed moderate positive correlations with the MRCI score, the strongest correlation being for gastro-intestinal medicines. 

### 3.4. Patient Perceived Burden

Of the 166 participants who completed the LMQ, a third (55; 33.1%) had LMQ-3 scores indicating a low level of perceived medicine burden, 53.6% (n = 89) had a moderate burden and 13.3% (n = 22) had a high burden.

The MRCI scores, number of medicines prescribed, maximum number of dosing times required per day, number of therapeutic groups and number of formulations all showed an increasing association with increasing burden. However, with the exception of the dosing frequency, the associations did not reach statistical significance (Table 4).

The LMQ-3 scores were significantly higher in patients prescribed drugs for neurological, gastro-intestinal and psychiatric conditions than in patients not prescribed drugs from these therapeutic groups (Table 5). Linear regression suggested that drugs for neurological conditions contributed most strongly to the LMQ scores.

Of the 22 patients with a high burden (LMQ score 110 or above), 21 (95%) used oral formulations, seven (32%) inhaled formulations, six (27%) used a topical formulation and another six (27%) used other formulations. Therefore, non-oral formulations were more common in this sub-group than in the overall population of 492 patients. The proportions of patients using medicines for central nervous system (73%), gastro-intestinal (45%), respiratory (36%), genito-urinary system (27%) and rheumatic conditions (27%) were all higher than in the general population. However, the small numbers do not permit the statistical evaluation of these findings.

## 4. Discussion

The findings provide some insight into the medicine burden in terms of the number, formulations and complexity of medicines prescribed for a general population using community pharmacies in England and also into the factors affecting patient perception of this burden. Four major therapeutic areas (cardiovascular, gastro-intestinal, respiratory and centrally-acting) constituted the large majority of prescribed medicines, and within three of these groups, patients were prescribed an average of at least two medicines, but could receive up to eight different cardiovascular and 11 centrally-acting medicines. 

Despite cardiovascular medicines being most commonly prescribed, they contributed less to medicine regimen complexity than all other therapeutic groups. This is most likely due to the MRCI scores taking into account the formulation and multiple daily dosing, since most cardiovascular medicines are oral solid doses requiring once daily administration. Unsurprisingly, products for respiratory, skin and eye conditions, which have both complex administration and require multiple daily dosing, resulted in the highest MRCI scores. 

The study confirmed that the overall number of medicines, frequency of dosing and the number of non-oral formulations contributed to patient-reported medicine burden. The number of different therapeutic groups was also found to be related to medicine burden and, therefore, the number of medical conditions is also likely to be important. The therapeutic groups which appeared to be most highly associated with patient-reported burden were gastro-intestinal and centrally-acting medicines, particularly those for neurological conditions. The finding that medicines for neurological conditions are associated with a higher burden is in line with a study in people with epilepsy which found that they experienced a significantly higher burden than in the general population (M. Cashin-Cox; unpublished data). Studies are required in patients with other neurological and psychiatric conditions to assess whether medicine burden is indeed high in these conditions. These studies should include chronic pain, since medicines for rheumatic conditions as well as centrally-acting medicines were more frequently found in those with the highest levels of burden. 

The number of medicines has been shown to increase with the number of medical conditions in a large cohort of Scottish patients [7], which found that cardiovascular conditions were associated with the greatest number of medicines. Our findings are in line with this, and with prescribing data for England [6], which show cardiovascular medicines to be the most commonly prescribed therapeutic group. However, our study also highlights the contribution of other types of conditions to both the number of medicines used and their complexity in individual patients. 

It is increasingly important to obtain the patient perspective on medicine burden and to learn more about its association with medicine-taking behaviour. Studies show that medicine complexity may be related to adherence [16], while small studies also indicate that some relationship exists between patient perceived burden and adherence [17,18]. These studies contrast others which have found that increasing the number of medicines prescribed has no effect on adherence and may thus be regarded by health professionals as not problematic. Two studies in hypertension found that medicine burden measured as the number of medicines was either unrelated to adherence [19] or that higher burden was positively associated with both persistence and the medication possession ratio [20]. The authors of the latter study suggested that concerns about medication burden should not deter clinicians from adding more medicines to an individual’s regimen. Another study found that doctors were more likely to initiate treatments for hypertension, diabetes and lipid-lowering drugs in patients already using more drugs for other chronic diseases, also concluding that medicine burden was not a barrier for initiating new medicines [21]. These studies show that the increasing burden of adding more and more medicines to an already onerous regimen on the patient has, in the past, not always been considered and, while adherence is an important measure of medicine-taking behavior, adherence may be achieved at the expense of issues which are potentially burdensome. An awareness of regimen complexity is also important when prescribing or reviewing medicines, since, as our study shows, the dosing frequency and other aspects of regimen complexity can add to the patient’s burden. 

This study is the first attempt to describe the types of medicines which may contribute to the highest levels of medicine burden and relates these to different aspects of regimen complexity, which may help to identify areas for further study. It provides a small snapshot of the patterns of medicines used in only one geographical location in England. Moreover, the number of patients for whom medicine burden could be compared to prescription regimen was small. Dispensed medicines may not equate to medicines actually taken, but are more likely to reflect this than prescribed medicines from medical records. No information on the actual medical conditions was available. Much more work is needed to assess the factors that influence the relationship between the number of medicines, regimen complexity, adherence and patient-perceived medicine burden.

## 5. Conclusions

While cardiovascular medicines constitute the highest number of medicines used, centrally-acting and gastro-intestinal medicines contribute more to both complexity and medicine burden. Larger studies are required to determine the medical conditions associated with greatest medicine burden. Based on the current findings, future work should seek to determine medicine burden in patients with specific medical conditions, especially mental health and neurological conditions, including chronic pain. 

## Figures and Tables

**Table 1:** Therapeutic classes of the medicines prescribed.

Therapeutic Group	Number of Prescriptions (% of 2700)	Number of Patients (% of 492)	Mean Number of Medicines per Patient (Median; Range)
Cardiovascular	813 (30.1%)	298 (60.6%)	2.7 (2; 1–8)
All central nervous system drugs	595 (22.0%)	265 (53.9%)	2.2 (2; 1–11)
Psychiatry	261 (9.7%)	182 (37.0%)	1.4 (1; 1–8)
Neurology	334 (12.4%)	185 (37.6%)	1.8 (1; 1–9)
Gastro-intestinal drugs	275 (10.2%)	189 (38.4%)	1.5 (1; 1–5)
Respiratory drugs	246 (9.1%)	127 (25.8%)	2.0 (2; 1–6)
All endocrine drugs	266 (9.9%)	185 (37.6%)	1.4 (1; 1–5)
Diabetes	124 (4.6%)	75 (15.2%)	1.7 (1; 1–5)
Other endocrine drugs	123 (4.6%)	111 (22.6%)	1.1 (1; 1–3)
Musculo-skeletal	95 (3.5%)	82 (16.7%)	1.2 (1; 1–3)
Urinary/sex hormones	63 (2.3%)	51 (10.4%)	1.2 (1; 1–3)
Skin products	143 (5.3%)	78 (15.9%)	1.8 (1; 1–10)
Eye products	42 (1.6%)	33 (6.7%)	1.3 (1; 1–2)
Other	125 (4.4%)	123 (24.2%)	1.3 (1; 1–4)

**Table 2:** Range of formulations prescribed.

Formulation Type	Number of Prescriptions (% of total)	Number of Patients (% of 492)
Oral solid dose	2132 (79.0%)	477 (97.0%)
Inhaler	192 (7.1%)	100 (20.3%)
Topical	183 (6.8%)	111 (22.6%)
Eye, ear and nasal	73 (2.7%)	59 (12.0%)
Other	120 (4.4%)	82 (16.7%)
Any formulation other than oral solid dose	568 (21.0%)	245 (49.8%)

**Table 3:** Relationships between the MRCI score and the therapeutic group.

Therapeutic Group	Results of Linear Regression Analysis			Correlations between MRCI Score and Number of Prescribed Medicines within Therapeutic Groups *	
	B	95% CI	*p* Value	R Value	*p* Value
Cardiovascular	5.20	3.55; 6.94	<0.001	0.35	<0.001
Respiratory	9.83	8.46;11.19	<0.001	0.34	<0.001
Gastro-intestinal	5.94	4.60; 7.27	<0.001	0.46	<0.001
Psychiatric	1.99	0.67; 3.30	0.003	0.31	<0.001
Neurological	5.53	4.13; 6.94	<0.001	0.39	<0.001
Endocrine	2.73	1.29; 4.18	<0.001	0.30	<0.001
Skin	7.41	5.74; 9.08	<0.001	0.34	0.002
Musculo-skeletal	3.39	1.72; 3.99	<0.001	Insufficient variation for analysis	

Note: * Spearman’s correlation coefficient.

**Table 4:** Association of the various aspects of complexity with the perceived medicine burden.

Complexity Measure	Perceived Medicine Burden (LMQ Category)			*p* Value *
	Low (55)	Moderate (89)	High (22)	
MRCI score	10.5 ± 10.7	12.0 ± 9.5	16.2 ± 11.4	0.088
Number of medicines	3.7 ± 3.5	4.4 ± 3.8	5.8 ± 4.1	0.075
Number of formulations	1.5 ± 0.7	1.6 ± 0.8	1.9 ± 1.0	0.14
Maximum number of daily doses	2.2 ± 1.2	2.5 ± 1.3	3.1 ± 1.4	0.029
Number of therapeutic groups	2.4 ± 1.5	2.7 ± 1.7	3.2 ± 1.6	0.175

Note: * Analysis of variance.

**Table 5:** Relationship between the therapeutic group and the perceived burden, measured by the LMQ-3 score.

Therapeutic Group	Results of Linear Regression Analysis			Mean LMQ-3 Score		
	B	95% CI	*p* Value	Present (n)	Not Present (n)	*p* Value *
Cardiovascular	−3.28	−9.66; 3.09	0.311	93.7 (103)	95.6 (64)	0.521
Respiratory	−0.02	−7.34; 7.30	0.996	97.4 (31)	93.7 (135)	0.319
Gastro-intestinal	5.19	−1.89; 12.27	0.150	100.2 (50)	91.8 (116)	0.007
Psychiatric	2.83	−3.60; 9.27	0.386	98.0 (59)	92.2 (126)	0.043
Neurological	7.05	0.07; 14.03	0.048	100.7 (55)	91.3 (130)	0.001
Endocrine	−3.37	−10.43; 3.68	0.346	94.0 (97)	94.5 (129)	0.618
Skin	−4.59	−13.31; 4.13	0.300	94.3 (21)	94.4 (145)	0.985
Musculo-skeletal	3.33	−4.79; 11.45	0.419	101.4 (28)	92.9 (138)	0.491

Note: * T-test.
